# Vascular Relaxation and Blood Pressure Lowering Effects of *Prunus mume* in Rats

**DOI:** 10.3390/bioengineering10010074

**Published:** 2023-01-06

**Authors:** Cheolmin Jo, Bumjung Kim, Kyungjin Lee, Ho-Young Choi

**Affiliations:** 1 Department of Herbal Pharmacology, College of Korean Medicine, Kyung Hee University, Seoul 02447, Republic of Korea; 2 Department of Oriental Health Management, Kyung Hee Cyber University, Seoul 02447, Republic of Korea

**Keywords:** *Prunus mume*, vasorelaxant, hypertension, angiotensin receptor blockers

## Abstract

*Prunus mume* Siebold et Zuccarini is mainly consumed as processed fruits in beverages, vinegar, alcohol, or fruit syrup; studies have reported various functional effects. Many pharmacological and functional studies exist on fruit extracts or processed foods using fruits, however, efficacy studies on various parts of *P. mume*, including the bark, branches, flowers, and leaves, have not been sufficiently conducted. A previous study revealed that a 70% ethanol extract of *P. mume* branches induced vascular endothelium-dependent vasorelaxant effects in rat thoracic aortic rings. Therefore, we hypothesized that various parts (the fruits, flowers, leaves, and bark) might have vasorelaxant effects. We evaluated the effects of *P. mume* extracts on the vascular relaxation of isolated rat thoracic aorta and hypotensive effects in spontaneous hypertensive rats (SHR). A 70% ethanol extract of *P. mume* bark (PBaE) was the most effective, thus, we investigated its vasorelaxant mechanisms and hypotensive effects. PBaE lowered the blood pressure in SHR and induced the vascular endothelium-dependent relaxation of isolated rat aortic rings via the NO/sGC/cGMP and the PGI_2_ pathways in the vascular smooth muscle. Potassium channels, such as K_Ca_, K_ATP_, K_V_, and K_ir_, were partially associated with a PBaE-induced vasorelaxation. Therefore, PBaE might help prevent and treat hypertension.

## 1. Introduction

Hypertension (high blood pressure) is a major cause of premature death worldwide, affecting one in four men and one in five women (over 1 billion people) [1]. The main causes of a high blood pressure are unhealthy eating habits, a lack of exercise, smoking, drinking, and obesity. Therefore, reducing these modifiable risk factors most effectively prevent and control high blood pressure [1]. However, in uncontrolled high blood pressure, despite these lifestyle changes, antihypertensive drugs, such as angiotensin-converting enzyme inhibitors, angiotensin receptor blockers, calcium channel blockers, renin inhibitors, thiazide diuretics, α-adrenergic blockers, β-adrenergic blockers, sympatholytic agents, and vasodilators are used [2]. Despite the research and discovery of these various drugs, the number of hypertensive patients is not decreasing, and there is an increasing demand for more efficient and reliable approaches to prevent and treat hypertension.

Natural products have been used to treat various diseases, including cancer and cardiovascular disease [3,4]. From 1946 to date, approved nature-derived cancer treatments account for over half of all anti-cancer drugs. In hypertension, natural products accounted for 20% of all approved antihypertensive drugs between 1981 and 2019 [5]. Therefore, natural products can treat and prevent hypertension.

*Prunus mume* Siebold et Zuccarini is a deciduous tree in the Rosaceae family with over 3000 years of cultivation history [6]. They are mainly used as landscape or fruit trees in Asia, including Korea. The fumigated fruit of *P. mume* is used as the traditional medicine, “Omae,” in Korea [7]. Additionally, various parts (the fruits, flowers, leaves, branches, seeds, and roots) of *P. mume* have been used in traditional Chinese medicine [8]. *P. mume* is mainly consumed as fruits processed into beverages [9,10,11], vinegar [12], alcohol [13,14], or fruit syrup [15]. Studies have identified their various functions. Additionally, flowers are used as tea [16].

Phytochemical research on *Prunus mume* revealed compounds such as phenols, organic acids, steroids, terpenes, lignans, benzyl glycosides, furfural, cyanogenic glycosides, and alkaloids, mainly in flowers and fruits [17]. In basic research, various activities, such as the antioxidant [18,19], anti-inflammatory [20], anti-cancer [21,22], anti-osteoporosis [23], anti-obesity [24], anti-helicobacter [25], blood flow improvement [26], anti-allergic [27], anti-fatigue [12], hepatoprotective [28], and immune-enhancing effects [29] of the fruits of *P. mume* have been identified. There are pharmacological and functional studies on fruit extracts or processed foods using fruits; nonetheless, the efficacy studies on various parts of *P. mume,* such as the bark, branches, flowers, and leaves, have not been sufficiently studied. Some processed fruit products help control blood pressure; however, the scientific evidence is lacking. Studies have revealed that *P. mume* fruit did not significantly impact the control of blood pressure in patients with hypertension [30].

However, we demonstrated in a previous study that a 70% ethanol extract of *P. mume* branches induced vascular endothelium-dependent vasorelaxant effects in rat thoracic aortic rings [7]. Therefore, we hypothesized that various parts of *P. mume* might have vasorelaxant effects. Therefore, to further validate the benefits of *P. mume* products in pharmaceutical and nutraceutical applications, the vasodilatory activity of various *P. mume* parts was investigated. Additionally, one extract with vasorelaxant effects was selected, and its vascular relaxation mechanism and hypotensive effects were evaluated.

## 2. Materials and Methods

### 2.1. Chemicals

Angiotensin II (Ang II), calcium chloride (CaCl_2_), phenylephrine hydrochloride (PE), NG-nitro-L-arginine methyl ester (L-NAME), 1H-[1,2,4]Oxadiazolo[4,3-a]quinoxalin-1-one (ODQ), methylene blue (MB), indomethacin, and ethylene glycol-bis(2-aminoethylether)-N, N, N′, N′-tetraacetic acid (EGTA) were purchased from Sigma Aldrich, Inc. (St. Louis, MO, USA). Tetraethylammonium (TEA), 4-aminopyridine (4-AP), and glibenclamide were purchased from Wako Pure Chemical Industries, Ltd. (Osaka, Japan). Magnesium sulfate (MgSO_4_), potassium chloride (KCl), and potassium phosphate monobasic (KH_2_PO_4_) were purchased from Duksan Pure Chemicals Co., Ltd. (Ansan, Korea). Barium chloride (BaCl_2_), glucose, sodium chloride (NaCl), sodium hydrogen carbonate (NaHCO_3_), and urethane were purchased from Daejung Chemicals & Metals Co., Ltd. (Siheung, Korea).

### 2.2. Plant Material and Extraction

The fresh fruits, flowers, leaves, branches, and bark of *Prunus mume* were collected from Dangjin-si, Chungcheongnam-do, the Republic of Korea, and the taxonomic identities of the plant were authenticated by a professor in the Department of Herbology, the University of Kyung Hee, the Republic of Korea. The collected plant parts were washed with water to remove contaminants, cut into small pieces, and dried in a convection oven. The dried samples are mixed with water or 70% ethanol and boiled for 2 h. After vacuum filtration, the filtrate was frozen at −20 °C and freeze-dried to obtain 10 extract powders (Table 1).

### 2.3. Animals

Male Sprague Dawley rats (SD, 220–250 g, 8 weeks old) were obtained from Daehanbiolink Co., Ltd. (Eumseong, Korea). Male spontaneously hypertensive rats (SHR, 200–250 g, 8 weeks old) were purchased from Charles River Laboratories (Yokohama, Japan). All animal procedures were conducted according to the animal welfare guidelines and were approved (KHSASP-21-050) by the Kyung Hee University Institutional Animal Care and Use Committee. The animals were maintained under controlled environmental conditions (12/12 h light/dark cycle, 22 ± 2 °C). Food and water were available ad libitum.

### 2.4. Measurement of Vasorelaxant Activity

#### 2.4.1. Preparation of Rat Aortic Rings

SD rats were anesthetized using urethane (1.2 g/kg, i.p.). After the abdominal incision was made to expose the aorta, the thoracic aorta was separated. The fat and connective tissues were removed while immersed in Krebs–Henseleit buffer (KH, composition (mM): NaCl, 118.0; KCl, 4.7; MgSO_4_, 1.2; KH_2_PO_4_, 1.2; CaCl_2_, 2.5; NaHCO_3_, 25.0; and glucose, 11.1; pH 7.4). The tissue bath solution bubbled continuously with 95% O_2_ and 5% CO_2_ at 37 °C. Aortic rings were made by cutting the thoracic aorta approximately 3 mm long, placing it between two stainless steel hooks in organ bath chambers, and connecting it to isometric force transducers. After incubation without tension for 20 min, the vessel segments were allowed to equilibrate for 40 min at a resting tension of 1.2 g. The KH was replaced every 20 min during the equilibrium period. Changes in the tension were recorded via the isometric transducers connected to a data acquisition system (PowerLab, ADI instrument Co., Ltd., New South Wales, Australia). Ca^2+^-free KH buffer was prepared by replacing CaCl_2_ with 1 mM EGTA.

#### 2.4.2. Vasorelaxant Effects of *Prunus mume* Extract on Isolated Aortic Rings

The aortic rings were pre-contracted with PE (1 μM). When the degree of contraction reached the maximum, *Prunus mume* flower water extract (PFlW), *Prunus mume* flower 70% ethanol extract (PFlE), *Prunus mume* fruit water extract (PFrW), *Prunus mume* fruit 70% ethanol extract (PFrE), *Prunus mume* leaf water extract (PLW), *Prunus mume* leaf 70% ethanol extract (PLE), *Prunus mume* branch water extract (PBrW), *Prunus mume* branch 70% ethanol extract (PBrE), *Prunus mume* bark water extract (PBaW), or *Prunus mume* bark 70% ethanol extract (PBaE) was added cumulatively (10–1000 μg/mL). To compare the effect of each extract using the concentration, the minimum concentration was 10 μg/mL, and the maximum concentration was 1000 μg/mL. However, the concentration is flexible and can be changed based on the degree of the blood vessel relaxation. Mechanism studies were conducted by selecting the extract with the greatest vasorelaxant effect.

The equation for calculating the degree of vasorelaxation is:

Relaxation (%) = [{(B − A) − (C − A)}/(B − A)] × 100

where A = the resting tension of aortic rings before pre-contraction with PE; B = the maximum contraction of aortic rings after pre-contraction using PE; and C = the contraction of the aortic rings after the drug treatment.

#### 2.4.3. Effect of PBaE on Endothelium-Intact and Endothelium-Denuded Aortic Rings

To investigate whether the vascular endothelium participates in the vasorelaxant mechanism of PBaE, we measured the vasorelaxant effect of PBaE (10 μg/mL) with or without vascular endothelium on aortic rings pre-contracted with PE (1 μM) KH buffer.

#### 2.4.4. Effect of PBaE on Endothelium-Intact Aortic Rings Pre-Incubated with L-NAME, Indomethacin, or Combination of L-NAME and Indomethacin

To determine the effect of PBaE on nitric oxide (NO), cyclooxygenase (COX), and prostacyclin (PGI_2_), the endothelium-intact aortic rings were pre-incubated with an inhibitor, such as L-NAME (NO synthase inhibitor, 100 μM), indomethacin (COX inhibitor, 10 μM), and L-NAME (100 μM) + indomethacin (10 μM), for 20 min before pre-contraction using PE (1 μM). The cumulative concentration–response of PBaE (0.5–10 μg/mL) on the aortic ring was compared to that of the control (not treated with inhibitors).

#### 2.4.5. Effect of PbaE on Endothelium-Intact Aortic Rings Pre-Incubated with ODQ or MB

To determine the effect of PbaE on soluble guanylate cyclase (sGC) or cyclic guanosine monophosphate (cGMP), the endothelium-intact aortic rings were pre-incubated with inhibitors, such as ODQ (sGC inhibitor, 10 μM) or MB (cGMP inhibitor, 10 μM), for 20 min before pre-contraction using PE (1 μM). The cumulative concentration–response of PbaE (0.5–10 μg/mL) on the aortic ring was compared to that of the control (not treated with inhibitors).

#### 2.4.6. Effect of PbaE on Endothelium-Intact Aortic Rings Pre-Incubated with TEA, Glibenclamide, 4-AP, or BaCl_2_

To examine the effect of PbaE on the non-selective calcium-activated K^+^ (K_Ca_), non-specific adenosine triphosphate-sensitive K^+^ (K_ATP_), voltage-dependent K^+^ (K_V_), and inwardly rectifying K^+^ (K_ir_) channel, the endothelium-intact aortic rings were pre-incubated with inhibitors, such as TEA (K_Ca_ blocker, 1 mM), glibenclamide (K_ATP_ blocker, 10 μM), 4-AP (K_V_ blocker, 1 mM), and BaCl_2_ (K_ir_ blocker, 10 μM), for 20 min before pre-contraction using PE (1 μM). The cumulative concentration–response of PbaE (0.5–10 μg/mL) on the aortic ring was compared to that of the control group (not treated with inhibitors).

#### 2.4.7. Effects of PbaE on Extracellular Ca^2+^-Induced Contraction

To investigate the mechanism of the vasorelaxant effects through the receptor-operated calcium channel (ROCC), the rat thoracic aortic ring was pretreated using PBaE (10 μg/mL) in Ca^2+^-free KH buffer, and PE was administered 10 min later to activate ROCC in the aortic rings. CaCl_2_ (0.3–10 mM) was administered to the aortic ring in which the calcium channel was activated, and the inhibitory effect of PBaE on the vasoconstriction induced by Ca^2+^ was measured.

#### 2.4.8. Inhibitory Effect of PBaE Pre-Treatment on Ang II-Induced Contraction

To investigate the vasorelaxant mechanism related to the angiotensin receptor, the aortic rings were pre-incubated with PBaE (10 μg/mL) for 20 min. Then, Ang II (10^−9^–10^−7^ M) was cumulatively administered to measure the inhibitory effect of PBaE on the Ang II-induced vasoconstriction.

### 2.5. Blood Pressure Measurement

**Figure 1:**
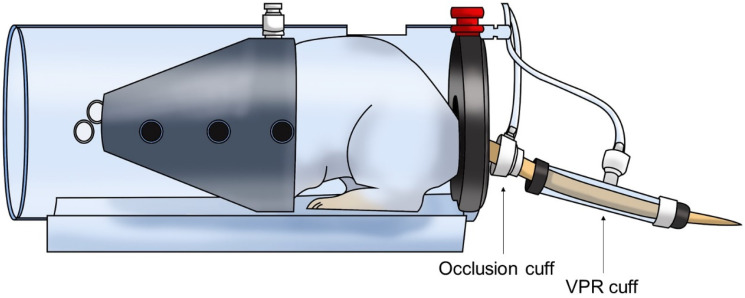
Schematic of the non-invasive tail-cuff method in rat.

The systolic blood pressure (SBP) and diastolic blood pressure (DBP) of the SHRs were measured using the non-invasive tail-cuff method (CODA 8-Channel High Throughput Noninvasive Blood Pressure System, Kent Scientific Co., Ltd., Torrington, CT, USA). Measurements were taken after restraining the animals with an adjustable nose cone holder to restrict excessive movement and a rear gate with access to the base of the animal’s tail. SBP and DBP of SHR were measured and recorded using an occlusion cuff and a volume pressure recording (VPR) cuff sensor (Figure 1). The 12 animals were randomly divided into three groups. Each group was orally administered PBaE (100 mg/kg), PBaE (300 mg/kg), and distilled water (control group). The blood pressure of the SHRs was measured before the administration and 1, 2, 4, and 8 h after the drug administration. During the experiment, the surface temperature of the animals was maintained at 32−35 °C using a heating pad.

### 2.6. Data Analysis

The values are expressed as the mean ± standard error of the mean (SEM) of n animals (for in vivo studies) or n aortic rings (for ex vivo studies). All data analyses were performed using GraphPad Prism 8 (GraphPad Software, San Diego, CA, USA). The concentration–response relationships were analyzed using an ordinary two-way analysis of variance followed by the Bonferroni’s test. Unpaired Student’s t-test was used for two group comparisons. A *p* < 0.05 was considered significant.

## 3. Results

### 3.1. Vasorelaxant Effects of PFrW and PFrE

**Figure 2:**
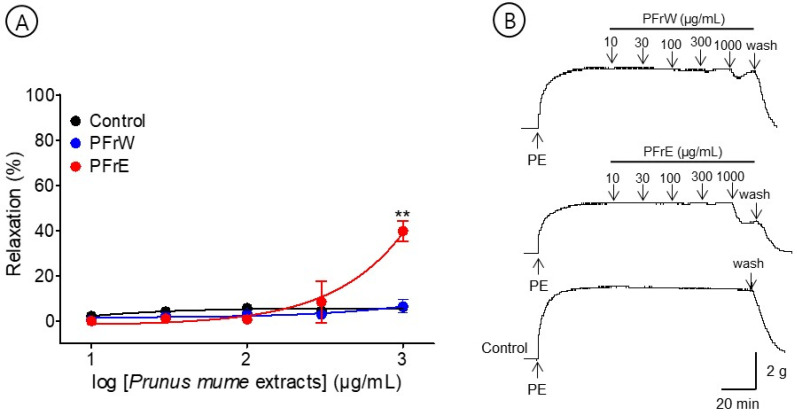
Cumulative concentration–response curves (**A**) and representative original traces (**B**) of *Prunus mume* fruit water extract (PFrW) and *Prunus mume* fruit 70% ethanol extract (PFrE) on endothelium-intact aortic rings. Relaxation was expressed as a percentage of phenylephrine hydrochloride (PE, 1 μM)-induced contraction. Values are expressed as mean ± SEM (n = 4–6). ** *p* < 0.01 vs. control.

The effects of PFrW (10–1000 μg/mL) and PFrE (10–1000 μg/mL) were compared to evaluate the vasorelaxant effect of the *P. mume* fruit extracts. PFrW did not significantly affect the aortic rings pre-contracted with PE (1 μM). Among them, PFrE caused a concentration-dependent relaxation on the endothelium-intact aortic ring. The half maximal effective concentration (EC_50_) and maximal relaxation (R_max_) were 363.8 ± 20.8 μg/mL and 37.0 ± 6.5%, respectively (Figure 2).

### 3.2. Vasorelaxant Effect of PFlW and PFlE

**Figure 3:**
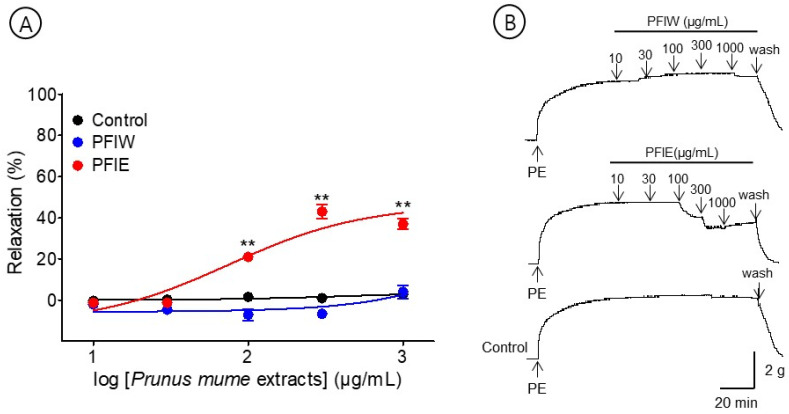
Cumulative concentration–response curves (**A**) and representative original traces (**B**) of *Prunus mume* flower water extract (PFlW) and *Prunus mume* flower 70% ethanol extract (PFlE) on endothelium-intact aortic rings. Relaxation was expressed as a percentage of phenylephrine hydrochloride (PE, 1 μM)-induced contraction. Values are expressed as mean ± SEM (n = 4–6). ** *p <* 0.01 vs. control.

The effects of PFlW (10–1000 μg/mL) and PFlE (10–1000 μg/mL) were compared to evaluate the vasorelaxant effect on the *P. mume* flower extracts. PFlW did not cause a significant effect on the aortic rings pre-contracted with PE (1 μM). Among them, PFlE caused the concentration-dependent relaxation of the endothelium-intact aortic ring. The EC_50_ and R_max_ were 96.5 ± 1.2 μg/mL and 33.5 ± 10.1%, respectively (Figure 3).

### 3.3. Vasorelaxant Effects of PLW and PLE

**Figure 4:**
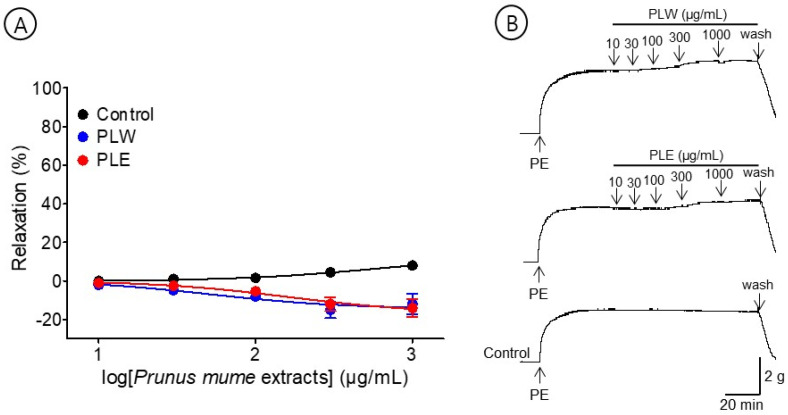
Cumulative concentration–response curves (**A**) and representative original traces (**B**) of *Prunus mume* leaf water extract (PLW) and *Prunus mume* leaf 70% ethanol extract (PLE) on endothelium-intact aortic rings. Relaxation was expressed as a percentage of phenylephrine hydrochloride (PE, 1 μM)-induced contraction. Values are expressed as mean ± SEM (n = 4–6).

The effects of PLW (10–1000 μg/mL) and PLE (10–1000 μg/mL) were compared to examine the vasorelaxant effect on the *P. mume* leaf extracts. PLW and PLE did not relax the pre-contracted aortic rings but caused a constriction at all concentrations (10–1000 μg/mL) (Figure 4).

### 3.4. Vasorelaxant Effects of PBrW and PBaW

**Figure 5:**
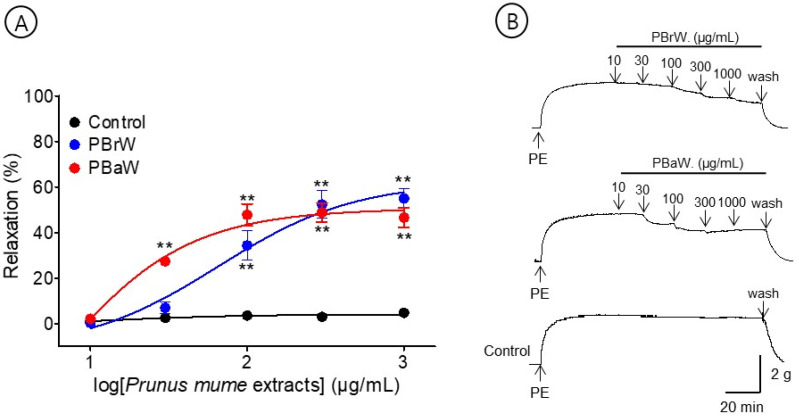
Cumulative concentration–response curves (**A**) and representative original traces (**B**) of *Prunus mume* branch water extract (PBrW) and *Prunus mume* bark water extract (PBaW) on endothelium-intact aortic rings. Relaxation was expressed as a percentage of phenylephrine hydrochloride (PE, 1 μM)-induced contraction. Values are expressed as mean ± SEM (n = 4–6). ** *p <* 0.01 vs. control.

To evaluate the effects of the branch and bark water extracts, we compared the vasorelaxant effects of PBrW (10–1000 μg/mL) and PBaW (10–1000 μg/mL). PBrW and PBaW caused the vasorelaxation of the endothelium-intact aortic rings pre-contracted with PE (1 μM). The EC_50_ and R_max_ for PBrW and PBaW were 78.4 ± 1.5 μg/mL and 55.0 ± 4.4% and 28.9 ± 1.0 μg/mL and 48.8 ± 4.1%, respectively (Figure 5).

### 3.5. Vasorelaxant Effects of PBrE and PBaE

**Figure 6:**
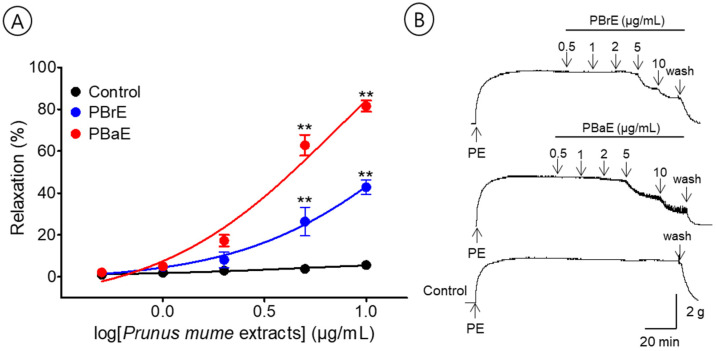
Cumulative concentration–response curves (**A**) and representative original traces (**B**) of *Prunus mume* branch 70% ethanol (PBrE) and *Prunus mume* bark 70% ethanol (PBaE) on endothelium-intact aortic rings. Relaxation was expressed as a percentage of phenylephrine hydrochloride (PE, 1 μM)-induced contraction. Values are expressed as mean ± SEM (n = 5–6). ** *p <* 0.01 vs. control.

To evaluate the effects of the branch and bark water extracts, we compared the vasorelaxant effects of PBrE (0.5–10 μg/mL) and PBaE (0.5–10 μg/mL). PBrE and PBaE caused the concentration-dependent relaxation of the endothelium-intact aortic ring pre-contracted with PE (1 μM). The EC_50_ and R_max_ for PBrE and PBaE were 4.0 ± 1.1 μg/mL and 42.8 ± 3.4% and 3.2 ± 1.0 μg/mL and 81.5 ± 2.7%, respectively (Figure 6).

### 3.6. Vasorelaxant Mechanism of PBaE

The PBaE was the most effective, therefore, it was investigated further for the mechanism of its vasorelaxant effect (Table 2). Mechanism studies were designed to evaluate whether the vasorelaxant effects of PBaE are related to the endothelium-dependent pathway, NO/sGC/cGMP pathway, PGI_2_ pathway, potassium channel, calcium channel, or angiotensin receptor.

#### 3.6.1. Vasorelaxant Effects of PBaE on Endothelium-Intact or Endothelium-Denuded Aortic Rings

**Figure 7:**
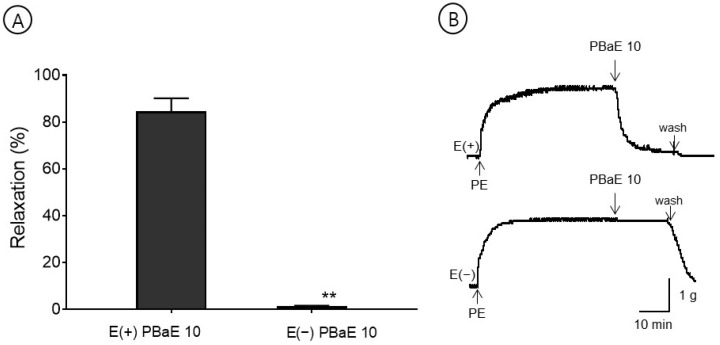
The vasorelaxant effect of *Prunus mume* bark in 70% ethanol (PBaE, 10 μg/mL) on intact [E(+)] or denuded [E(−)] endothelium rat aortic rings pre-contracted with phenylephrine hydrochloride (PE, 1 μM) (**A**). Representative traces under the indicated conditions (**B**). Values are expressed as mean ± SEM (n = 4–6). ** *p <* 0.01 vs. control.

The maximum relaxation effect concentration of PBaE, 10 μg/mL, was used in this experiment. PBaE (10 μg/mL) caused the vascular relaxation of the endothelium-intact aortic rings but did not induce the vascular relaxation of the endothelium-denuded aortic rings. The vasorelaxant effect in the PE-induced contraction was 84.5 ± 5.6% and 1.3 ± 0.3% for the endothelium-intact and endothelium-denuded aortic rings using 10 μg/mL, respectively (Figure 7).

#### 3.6.2. Vasorelaxant Effect of PBaE on Endothelium-Intact Aortic Rings Pre-Incubated with L-NAME, Indomethacin, or L-NAME and Indomethacin Combined

**Figure 8:**
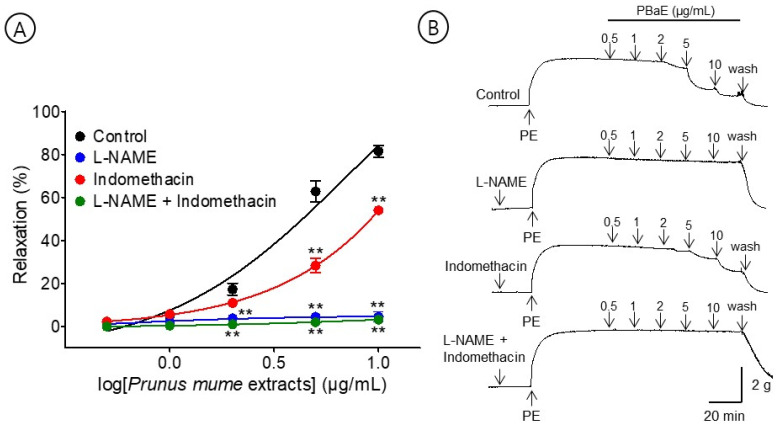
Cumulative concentration–response curves (**A**) and representative original traces (**B**) of vasorelaxant effect for *Prunus mume* bark in 70% ethanol (PBaE, 0.5–10 μg/mL) in the absence (control) or presence of NG-nitro-L-arginine methyl ester (L-NAME (100 μM), indomethacin (10 μM), or L-NAME (100 μM) + indomethacin (10 μM). Values are expressed as mean ± SEM (n = 4–6). ** *p* < 0.01 vs. control.

A pre-incubation with L-NAME (100 μM) significantly decreased the PBaE-induced relaxation of endothelium-intact aortic rings pre-contracted using PE (1 μM). In the presence and absence of L-NAME, the maximum relaxation effect was 4.5 ± 2.3% and 81.5 ± 2.7%, respectively (Figure 8). A pre-incubation with indomethacin (10 μM) significantly decreased the PBaE-induced relaxation of the endothelium-intact aortic rings pre-contracted using PE (1 μM). With and without indomethacin, the maximum relaxation effect was 54.0 ± 1.9% and 81.5 ± 2.7%, respectively (Figure 8). A pre-incubation with L-NAME (100 μM) + indomethacin (10 μM) significantly decreased the PBaE-induced relaxation of endothelium-intact aortic rings pre-contracted with PE (1 μM). In the presence and absence of L-NAME (100 μM) + indomethacin (10 μM), the maximum relaxation effect was 3.2 ± 0.9% and 81.5 ± 2.7%, respectively (Figure 8).

#### 3.6.3. Vasorelaxant Effect of PBaE on Endothelium-Intact Aortic Rings Pre-Incubated with ODQ or MB

**Figure 9:**
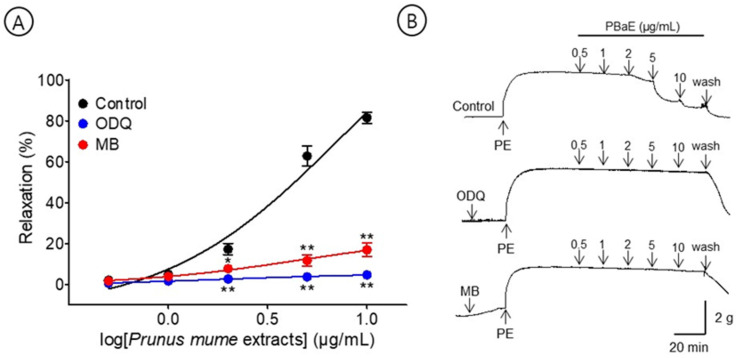
Cumulative concentration–response curves (**A**) and representative original traces (**B**) of vasorelaxant effect for *Prunus mume* bark in 70% ethanol (PBaE, 0.5–10 μg/mL) with (control) or without 1H-[1,2,4]Oxadiazolo [4,3-a]quinoxalin-1-one (ODQ, 10 μM) or methylene blue (MB, 10 μM). Values are expressed as mean ± SEM (n = 4–6). * *p* < 0.05, ** *p* < 0.01 vs. control.

A pre-incubation with MB (10 μM) significantly decreased the PBaE-induced relaxation of endothelium-intact aortic rings pre-contracted using PE (1 μM). In the presence and absence of ODQ, the maximum relaxation effect was 4.7 ± 0.4% and 81.5 ± 2.7%, respectively. A pre-incubation with MB (10 μM) significantly decreased the PBaE-induced relaxation of endothelium-intact aortic rings pre-contracted with PE (1 μM). In the presence and absence of MB, the maximum relaxation effect was 17.0 ± 3.4% and 81.5 ± 2.7%, respectively (Figure 9).

#### 3.6.4. Vasorelaxant Effect of PBaE on Endothelium-Intact Aortic Rings Pre-Incubated with TEA, Glibenclamide, 4-AP, or BaCl_2_

**Figure 10:**
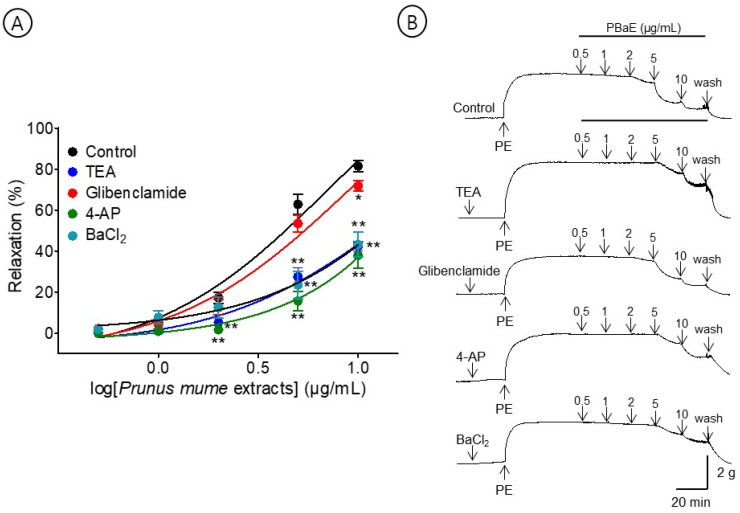
Cumulative concentration–response curves (**A**) and representative original traces (**B**) of vasorelaxant effect for *Prunus mume* bark in 70% ethanol (PBaE, 0.5–10 μg/mL) without (control) or with tetraethylammonium (TEA; 1 mM), glibenclamide (10 μM), 4-aminopyridine (4-AP; 1 mM), or barium chloride (BaCl_2,_ 10 μM). Values are expressed as mean ± SEM (n = 4–6). * *p* < 0.05, ** *p* < 0.01 vs. control.

A pre-incubation with potassium channel blockers, such as TEA, glibenclamide, 4-AP, and BaCl_2_, significantly decreased the PBaE-induced relaxation on endothelium-intact aortic rings pre-contracted with PE (1 μM). Using TEA (1 mM), glibenclamide (10 μM), 4-AP (1 mM), or BaCl_2_ (10 μM), the maximum relaxation effects were 42.5 ± 2.3%, 71.7 ± 2.5%, 38.0 ± 6.4, or 43.2 ± 6.1 at the 10 μg/mL, respectively (Figure 10).

#### 3.6.5. Vasorelaxant Effect of PBaE on Extracellular Ca^2+^-Induced Contraction

**Figure 11:**
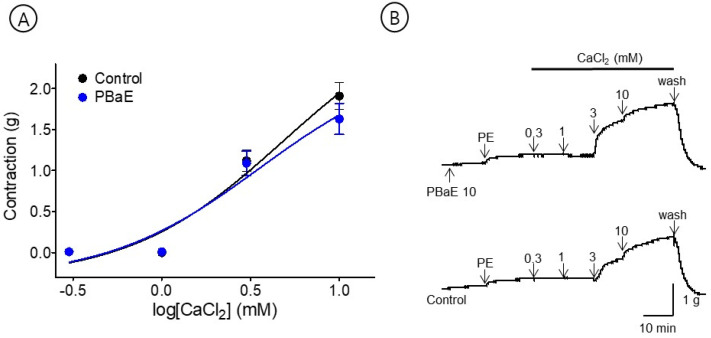
Inhibitory effect of *Prunus mume* bark in 70% ethanol (PBaE, 10 μg/mL) on the contraction induced by extracellular calcium chloride (CaCl_2_, 0.3–10 mM) on aortic rings that were pre-contracted with phenylephrine hydrochloride (PE, 1 μM) (**A**). Representative traces under the indicated conditions (**B**). Values are expressed as mean ± SEM (n = 4).

The cumulative addition of CaCl_2_ (0.3–10 mM) gradually contracted the tension of the aortic rings pretreated with PE (1 μM) in the Ca^2+^-free KH buffer. However, the PBaE (10 μg/mL) pre-treatment did not significantly differ from the control group (Figure 11).

#### 3.6.6. Inhibitory Effect of PBaE Pre-Treatment on Ang II-Induced Contraction

**Figure 12:**
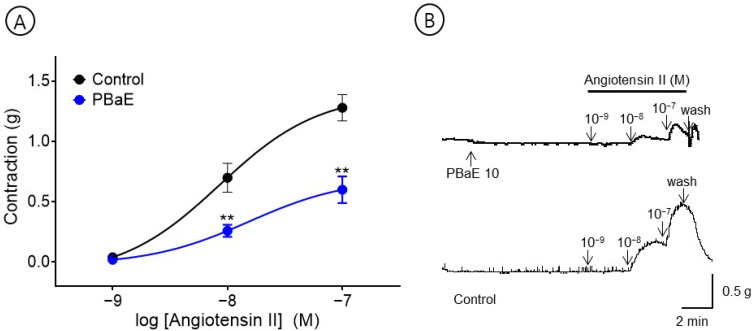
Inhibitory effect (**A**) and original representative traces (**B**) of *Prunus mume* bark in 70% ethanol (PBaE, 10 μg/mL) in the contraction induced by angiotensin II (Ang II, 10^−9^–10^−7^ M) on endothelium-intact aortic rings. Values are expressed as mean ± SEM (n = 6). ** *p* < 0.01 vs. control.

An experiment was performed to evaluate the inhibitory effect of the PBaE (10 μg/mL) on Ang II (10^−9^–10^−7^ M)-induced vasoconstriction in the endothelium-intact aortic rings. The PBaE pre-treatment significantly reduced the Ang II-induced contractions. The degree of contraction decreased to 0.60 ± 0.11 g compared to the control group and 1.28 ± 0.11 g at Ang II 10^−7^ M concentration, respectively (Figure 12).

### 3.7. Hypotensive Effect of PBaE on Blood Pressure in SHR

**Figure 13:**
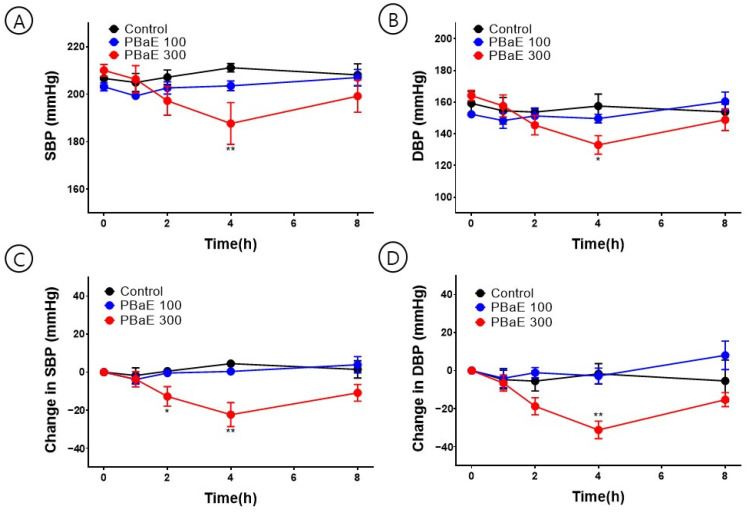
Hypotensive effect of *Prunus mume* in 70% ethanol extract (PBaE) in a spontaneously hypertensive rat (SHR). Systolic blood pressure (SBP) and diastolic blood pressure (DBP) were measured using the non-invasive tail-cuff system (**A**,**B**). Changes in SBP (**C**) and DBP (**D**) caused SHR by administering water (control), PBaE (100 mg/kg), or PBaE (300 mg/kg). The values are expressed as the mean ± SEM (n = 4). * *p* < 0.05, ** *p* < 0.01 vs. control.

To investigate the hypotensive effect of PBaE, SBP and DBP were measured 1, 2, 4, and 8 h after administering 100 or 300 mg/Kg of PBaE orally to SHR. At 4 h after administering PBaE 300 mg/kg, the SBP was significantly lowered from 210.0 ± 2.4 mmHg to 187.6 ± 8.7 mmHg, and the DBP decreased from 164.1 ± 3.2 mmHg to 133.0 ± 5.8 mmHg (Figure 13). Due to the characteristics of SHR, there was a difference in the blood pressure for each rat. A significant trend was confirmed by comparing the individual blood pressure values (Table 3).

## 4. Discussion

In this study, the fruits, flowers, leaves, branches, and bark of the *P. mume* were collected, and the extracts were prepared using two solvents: water and 70% ethanol. According to the results of the investigations on its vasorelaxant effects, 70% ethanol extracts of fruit, flower, branches, and bark had a vasorelaxant activity. Among them, branch and bark water extracts also had a vasorelaxant effect. However, the leaf water extract and 70% ethanol extract caused a vasoconstriction. The results reveal that the solvents of different polarities can extract various biologically active compounds, demonstrating a difference in the biological activity of each part, even within the same plant. Except for PBrW and PBaW, PFrE, PFlE, PBrE, and PBaE exhibited a vasorelaxant activity, suggesting that using an organic solvent, such as ethanol, to extract specific active ingredients that induce vasodilation may be advantageous. Among them, the vasorelaxant effects of PBrE and PBaE were 42.8 ± 3.4% and 81.5 ± 2.7%, respectively, at a relatively low concentration of 10 μg/mL, exhibiting strong vasorelaxant effects. Considering that the branch and bark of *P. mume* were not a single compound but a natural product consisting of a mixture of various compounds, it was a very effective vasodilator for regulating the tone of the blood vessels. In a previous study, amlodipine, a representative calcium channel blocker for treating hypertension, had a vasorelaxant effect of up to 48.6 ± 3.5% at 10 μg/mL [31]. Based on the vasodilation screening, PBaE was the most potent vasorelaxant, and a vasorelaxant mechanism and hypotensive effect study were performed on PBaE. The mechanism studies evaluated whether the vasorelaxant effect of PBaE was related to the endothelium-dependent pathway, NO/sGC/cGMP pathway, PGI_2_ pathway, potassium channel, calcium channel, or angiotensin receptor.

The vascular endothelium lies at the border between circulating blood cells and vascular smooth muscle cells and is essential in regulating the blood flow and vascular tone [32]. Vascular endothelium can synthesize and release different vasodilators, such as NO, PGI_2_, and the endothelium-derived hyperpolarizing factor [33,34]. The release of these substances causes an endothelium-dependent vasorelaxation in the rat thoracic aorta [35,36]. NO is produced from L-arginine in vascular endothelial cells under the catalysis of NO synthase and activates sGC to induce a cGMP-mediated vasodilation [37,38]. Additionally, PGI_2_ is generated from arachidonic acid by the catalytic action of COX and increases the cyclic adenosine monophosphate levels through an adenylate cyclase activation to induce vasodilation [39]. In the present study, PBaE induced vasorelaxation in endothelium-intact aortic rings pre-contracted with PE; however, this relaxation was significantly abrogated by removing the vascular endothelium. These results indicate that PBaE acts on vascular endothelial cells to stimulate vasodilators to mediate its endothelium-dependent vasorelaxation. Additionally, the endothelium-dependent vasorelaxation of PBaE was investigated using inhibitors, such as L-NAME (NO synthase inhibitor), ODQ (sGC inhibitor), MB (cGMP inhibitor), or indomethacin (COX inhibitor). The vasorelaxant effects of PBaE were significantly reduced by indomethacin and significantly inhibited by L-NAME, ODQ, MB, or L-NAME and indomethacin combined. Therefore, the results revealed that the PBaE vasodilation was mainly exerted through the NO/sGC/cGMP and PGI_2_ pathways.

The potassium channels also play a vital role in regulating the muscle contraction and vascular tone [40]. Four types of potassium channels exist in the arterial smooth muscle: K_Ca_, K_ATP_, K_V_, and K_ir_ channels. The activation of the potassium channels in vascular smooth muscle cells causes vasodilation by hyperpolarizing the cell membrane due to the efflux of K^+^ [41]. The results of this study revealed that the PBaE-induced relaxation in endothelium-intact aortic rings was reduced by the treatment with potassium channel blockers, including TEA, glibenclamide, 4-AP, and BaCl_2_. These data indicate that potassium channel activation in the vascular smooth muscle and endothelium, including K_Ca_, K_AT_P, K_V_, and K_ir_ channels, which may involve a PBaE-induced vasorelaxation.

Ang II is a final product of the renin-angiotensin system, which causes a vasoconstriction and increases the blood pressure by binding to the angiotensin receptor type 1 (AT-1) [42]. Therefore, the blood pressure and vascular tone can be controlled by using Ang II receptor blockers (ARB) that block Ang II from binding to AT-1 [43] or using angiotensin-converting enzyme inhibitors that inhibit the production of Ang II by directly acting on the converting enzyme [44]. Our results revealed that PBaE significantly reduced the degree of contractility induced by Ang II (10^−9^–10^−7^ M) by almost 50% in the endothelium-intact aortic rings. This suggests that PBaE replaces the function of ARB, inhibiting Ang II from binding to the angiotensin receptor. However, future studies are needed for more precise mechanisms by which PBaE contributes to regulating the vasoconstriction.

To evaluate the hypotensive effect of PBaE, the SBP and DBP of SHR were measured using the non-invasive tail-cuff method. They significantly decreased 4 h after the oral administration of PBaE (300 mg/kg).

In the present study, PBaE lowered the blood pressure in SHR and induced the vascular endothelium-dependent relaxation of isolated rat aortic rings via the NO/sGC/cGMP and PGI_2_ pathway mechanisms in the vascular smooth muscle. In addition, the potassium channels, such as K_Ca_, K_ATP_, K_V_, and K_ir_ channels, were partially associated with a PBaE-induced vasorelaxation. Therefore, PBaE can be developed as food or medicine to help prevent or treat high blood pressure. However, in this study, the changes in the blood pressures of the SHR were only measured for 8 h using a tail-cuff experiment to assess the antihypertensive effect of PBaE.

## 5. Conclusions

In conclusion, the vasorelaxant effect of PBaE was endothelium-dependent and was related to the NO/sGC/cGMP vascular prostacyclin pathway. In addition, potassium channels, such as K_Ca_, K_ATP_, K_V_, and K_ir_, were partially related to the PBaE-induced vasorelaxation. PBaE was effective in relaxing the contraction induced by Ang II, and the vasorelaxant effects of PBaE were unassociated with the influx of extracellular Ca^2+^ via ROCC. Furthermore, the SBP and DBP of SHR significantly decreased 4 h after the oral administration of PBaE (300 mg/kg). Our findings provide a basis for the use of the bark of *Prunus mume* as a medicinal and food resource. In future studies, the comparative evaluation of non-polar solvent extracts and safety and stability analyses, including the identification and standardization of the active ingredients, the determination of the appropriate dose, and a toxicity evaluation, should be conducted.

## 6. Patents

On 10 June 2021, a patent was registered for composition for preventing and/or treating a hypertensive disease comprising an extract of *Prunus mume* Siebold et Zuccarini or a fraction thereof as an active ingredient (Registration number: 10-2265786).

## Figures and Tables

**Table 1:** List of *Prunus mume* extracts used in this study.

Plant Part	Collection Date	Extracts	Yield (%)	Abbreviation
Fruit	June 2020	Water	21.5	PFrW
Fruit	June 2020	70% Ethanol	31.5	PFrE
Flower	March 2020	Water	25.0	PFlW
Flower	March 2020	70% Ethanol	9.3	PFlE
Leaf	June 2020	Water	31.0	PLW
Leaf	June 2020	70% Ethanol	23.0	PLE
Branch	February 2020	Water	8.4	PBrW
Branch	February 2020	70% Ethanol	9.3	PBrE
Bark	February 2020	Water	23.0	PBaW
Bark	February 2020	70% Ethanol	20.0	PBaE

**Table 2:** EC_50_ and R_max_ of *Prunus mume* extract-induced vasorelaxation.

Samples	R_max_ (%)	EC_50_ (μg/mL)
PFrW	6.5 ± 2.9	-
PFrE	37.0 ± 6.5 **	363.8 ± 20.8
PFlW	4.0 ± 3.1	-
PFlE	33.5 ± 10.1 **	96.5 ± 1.2
PLW	−12.0 ± 5.2	-
PLE	−14.0 ±4.6	-
PBrW	55.0 ± 4.4 **	78.4 ± 1.5
PBrE	42.8 ± 3.4 **	4.0 ± 1.1
PBaW	48.8 ± 4.1 **	28.9 ±1.0
PBaE	81.5 ± 2.7 **	3.2 ± 1.0

Values are expressed as mean ± SEM (n = 4–6). ** *p <* 0.01 vs. control. EC_50_, half maximal effective concentration; R_max_, maximal relaxation; PFrW, *Prunus mume* fruit water extract; PFrE, *Prunus mume* fruit 70% ethanol extract; PFlW, *Prunus mume* flower water extract; PFlE, *Prunus mume* flower 70% ethanol extract; PLW, *Prunus mume* leaf water extract; PLE, *Prunus mume* flower 70% ethanol extract; PBrW, *Prunus mume* branch water extract; PBaW, *Prunus mume* bark water extract; PBrE, *Prunus mume* branch 70% ethanol extract; PBaE, *Prunus mume* bark 70% ethanol extract.

**Table 3:** Effect of PBaE on blood pressure in SHR.

	Time (h)				
	0	1	2	4	8
Systolic blood pressure (mmHg)					
Control	206.6 ± 2.8	204.9 ± 3.7	207.1 ± 3.0	211.1 ± 1.7	208.1 ± 4.6
PBaE 100 mg/kg	203.1 ± 1.7	199.2 ± 1.3	202.6 ± 2.5	203.5 ± 2.0	207.0 ± 3.4
PBaE 300 mg/kg	210.0 ± 2.4	206.3 ± 5.7	197.2 ± 6.0	187.6 ± 8.7 **	199.1 ± 6.7
Diastolic blood pressure (mmHg)					
Control	159.2 ± 7.3	154.5 ± 8.4	153.7 ± 2.5	157.5 ± 7.5	153.8 ± 4.9
PBaE 100 mg/kg	152.3 ± 1.9	148.3 ± 4.9	151.2 ± 4.6	149.6 ± 2.6	160.4 ± 5.9
PBaE 300 mg/kg	164.1 ± 3.2	157.6 ± 6.9	145.4 ± 6.1	133.0 ± 5.8 *	148.8 ± 6.8

Values are expressed as mean ± SEM (n = 4). * *p* < 0.05, ** *p* < 0.01 vs. control. PBaE, *Prunus mume* bark 70% ethanol extract; SHR, spontaneously hypertensive rat.

## Data Availability

Not applicable.
